# A Phase Ib study of neoadjuvant immune biomarker modulation with cetuximab and motolimod in head and neck cancer (HNC)

**DOI:** 10.1186/2051-1426-3-S2-P144

**Published:** 2015-11-04

**Authors:** Robert L Ferris, Benjamin A Kansy, Sandra Poveda Gibson, Raghvendra M Srivastava, James Kyle Bryan, Robert M Hershberg

**Affiliations:** 1 University of Pittsburgh Cancer Institute, Pittsburgh, PA, USA; 2 Department of Otorhinolaryngology, University Hospital Essen, Germany; University of Pittsburgh Cancer Institute, Pittsburgh, PA, USA; 3 VentiRx Pharmaceuticals, Seattle, WA, USA

## Background

HNC is immunosuppressive, with low absolute lymphocyte counts, impaired activity of effectors such as natural killer cells (NK), and poor antigen-presenting function. The anti-EGFR monoclonal antibody cetuximab (CTX) prolongs survival in HNC, but only in a subset of patients. The clinical activity of CTX has been mechanistically linked to NK-mediated antibody-dependent cellular cytotoxicity (ADCC). However, recent data has shown that CTX increases the frequency of intratumoral CTLA-4^+^FoxP3^+^ regulatory T cells (Treg), which suppress ADCC and are associated with poor clinical outcome. In ex vivo experiments, this effect could be attenuated by targeting CTLA-4 on Tregs. Therefore it is possible that the clinical efficacy of CTX may be improved by strategies to enhance the activation of the immune system or inhibit the Treg suppressive effects.

Motolimod (formerly VTX-2337) is a novel Toll-like receptor 8 (TLR8) agonist which stimulates myeloid dendritic cells (mDC), monocytes, and NK. Preclinical data have demonstrated enhanced CTX and NK-mediated lysis of HNC cells and dendritic cross-priming of EGFR-specific CD8+ T cells. CTX and motolimod in HNC patients was tolerable and active in a Phase Ib study, with enhanced mobilization and activation of NK cells. The central hypothesis in this study is that NK and monocyte/mDC activation by CTX is enhanced by concomitant administration of motolimod, thereby amplifying the innate and adaptive immune response in the circulation and in the tumor microenvironment.

## Methods

This is a prospective Phase Ib clinical trial (NCT02124850) of preoperative treatment with CTX and motolimod. The primary objective is to determine the extent to which the administration of neoadjuvant CTX plus motolimod modulates immune biomarkers in peripheral blood and HNC tumors. An exploratory objective of the study is to assess whether this modulation of biomarkers can predict anti-tumor response. Subjects must have HNC stage II– IVA and be surgical candidates. CTX and motolimod will be administered for a 3–4 week preoperative period. The biomarker modulation and biopsies of skin/acneiform rash will be studied in correlation with clinical response by CT or MRI scan. Tumor apoptosis/proliferation will be assessed by biopsy pre- and post-treatment with CTX/motolimod. All patients will receive definitive surgical resection. Enrollment target is fifteen complete specimens (tumor, peripheral blood mononuclear cells and serum). A pending amendment will assess the impact of adding checkpoint inhibition to the CTX/motolimod combination.

